# Three-Directional Evaluation of Mitral Flow in the Rat Heart by Phase-Contrast Cardiovascular Magnetic Resonance

**DOI:** 10.1371/journal.pone.0150536

**Published:** 2016-03-01

**Authors:** Kristine Skårdal, Emil KS Espe, Lili Zhang, Jan Magnus Aronsen, Ivar Sjaastad

**Affiliations:** 1 Institute for Experimental Medical Research, Oslo University Hospital and University of Oslo, Oslo, Norway; 2 KG Jebsen Cardiac Research Center and Center for Heart Failure Research, University of Oslo, Oslo, Norway; 3 Bjørknes College, Oslo, Norway; University of California San Diego, UNITED STATES

## Abstract

**Purpose:**

Determination of mitral flow is an important aspect in assessment of cardiac function. Traditionally, mitral flow is measured by Doppler echocardiography which suffers from several challenges, particularly related to the direction and the spatial inhomogeneity of flow. These challenges are especially prominent in rodents. The purpose of this study was to establish a cardiovascular magnetic resonance (CMR) protocol for evaluation of three-directional mitral flow in a rodent model of cardiac disease.

**Materials and Methods:**

Three-directional mitral flow were evaluated by phase contrast CMR (PC-CMR) in rats with aortic banding (AB) (N = 7) and sham-operated controls (N = 7). Peak mitral flow and deceleration rate from PC-CMR was compared to conventional Doppler echocardiography. The accuracy of PC-CMR was investigated by comparison of spatiotemporally integrated mitral flow with left ventricular stroke volume assessed by cine CMR.

**Results:**

PC-CMR portrayed the spatial distribution of mitral flow and flow direction in the atrioventricular plane throughout diastole. Both PC-CMR and echocardiography demonstrated increased peak mitral flow velocity and higher deceleration rate in AB compared to sham. Comparison with cine CMR revealed that PC-CMR measured mitral flow with excellent accuracy. Echocardiography presented significantly lower values of flow compared to PC-CMR.

**Conclusions:**

For the first time, we show that PC-CMR offers accurate evaluation of three-directional mitral blood flow in rodents. The method successfully detects alterations in the mitral flow pattern in response to cardiac disease and provides novel insight into the characteristics of mitral flow.

## Introduction

Mitral flow is an important entity in cardiac diseases involving diastolic dysfunction and heart failure. The mitral flow in diastole is a result of two main components; the initial passive filling, E, caused by the depressurization of the ventricle, and the active filling, A, resulting from atrial contraction. The flow pattern depends on the loading conditions of the heart, thus in cardiac disease, the flow pattern is affected and changing in response to disease progression. Accordingly, evaluation of the mitral flow pattern is an important part of assessing cardiac function.

Traditionally, mitral flow is evaluated using pulsed Doppler echocardiography, relying on positioning a finite sampling volume at the assumed point of peak flow [1,2]. Since only the flow component directed directly towards or away from the transducer is detected, geometric correction is essential, but might be challenging, especially in small animals [3]. In addition, the spatial mitral flow velocity profile is inhomogeneous across the valve [4,5].

Cardiovascular magnetic resonance (CMR) has successfully been applied to assess blood flow in the major vessels in humans, and to observe regurgitation [6–8]. In the setting of mitral flow, three-directional velocity encoded phase-contrast CMR (PC-CMR) can provide classical flow parameters, such as peak mitral flow velocity and deceleration rate of the mitral flow signal, as well as directional and distributional assessment of the mitral flow. Additionally, profiting from complete spatial and temporal coverage of the flow profile, PC-CMR can be used to measure the blood volume flowing through the mitral valve [9].

In the present study, we have established and validated a PC-CMR protocol for studying three-directional time-resolved mitral inflow in rats. PC-CMR offers comprehensive evaluation of mitral flow with new insight into its directional characteristics and spatial distribution in healthy and diseased hearts.

## Materials and Methods

### Animal model

All procedures were approved by the Norwegian Animal Research Authority (FOTS ID 3820), in accordance with the *European Convention for the Protection of Vertebrate Animals used for Experimental and other Scientific Purposes* (ETS no.123). Male Wistar Hannover rats (~160g, Taconic Biosciences, NY, USA) were housed according to Appendix A of ETS no. 123, including a 12h/12h light/dark cycle, and *ad libitum* access to food and water. The rats underwent either aortic banding (AB) of the ascending aorta or a sham procedure as previously described [10,11]. After 6 weeks following AB or sham procedure, 7 sham and 7 AB rats underwent echocardiography and CMR, before *ex vivo* methods.

### Echocardiography

Echocardiography was performed using a Vevo2100 system (FUJIFILM VisualSonics Inc., Canada), evaluating both cardiac geometry and function, including mitral flow, as previously described [12]. Rats were anesthetized in a sedation chamber containing 96% O_2_ and 4% isoflurane before mask ventilation with a mixture of 98.25% O_2_ and 1.75% isoflurane. Left ventricular (LV) and left atrial diameter were assessed using M-mode in the parasternal long axis view. Mitral flow was measured using angle corrected pulsed wave Doppler sonography with the sampling volume placed at the tip of the mitral leaflets. Peak mitral flow velocity and deceleration rate of the mitral flow profile was analyzed off-line with the Vevo2100 system software.

### Cardiovascular magnetic resonance

CMR was performed using a 9.4 T dedicated small animal imaging system (Agilent Technologies Inc., USA) as previously described [13]. Rats were anesthetized in a sedation chamber containing 96% O_2_ and 4% isoflurane before mask ventilation with a mixture of 98.75–98.25% O_2_ and 1.25–1.75% isoflurane, and positioned prone in a dedicated animal bed. Temperature, respiration and ECG were continuously monitored and maintained during examination. A previously developed three-directional velocity encoded PC-CMR sequence [13,14] was optimized for assessment of mitral blood flow and applied to acquire a single imaging slice directly adjacent and parallel to the atrioventricular plane (repetition time 2.8ms, echo time 2.1ms, velocity encoding range ±1.39m/s, field of view 50mm×50mm, matrix 96×96 zero filled to 192×192, slice thickness 1.5mm and flip angle 7°). To ensure complete temporal coverage of the cardiac cycle, the R-R interval was oversampled, repeating the sequence every other R-peak. Depending on the heart rate, total acquisition time was 7–9 minutes, Additionally, 9–10 short-axis slices covering the whole LV was acquired using a gradient echo cine CMR protocol (repetition time 2.8ms, echo time 2.0ms, field of view 50mm×50mm, matrix 96×96 zero filled to 192×192, slice thickness 1.5mm and flip angle 15°). 47 temporal frames were acquired, resulting in a total scan time of 12–15 minutes. Both CMR protocols were ECG triggered and respiratory gated, and the temporal resolution in the resulting datasets was equal to the repetition time.

### CMR post-processing

From the cine CMR data, end-diastolic and end-systolic volumes (EDV and ESV), stroke volume (SV), ejection fraction (EF) and LV mass were calculated [15]. From the PC-CMR data, three-dimensional velocity vectors were reconstructed pixel-wise as previously described [13]. The reconstruction process involved compensation of baseline phase offsets [16]. The area of mitral flow was semi-automatically determined applying a variant of the method of Bollache et al. [7]. In short, we segmented the mitral valve orifice throughout diastole by combining detection of flow direction with identification of its largest connected area in an automatic fashion. The process was commenced in a manually defined diastolic phase of the cardiac cycle. Then, the area of diastolic flow was traced backwards and forwards until no flow of the inflowing direction was detected, resulting in a binary mask identifying mitral flow area only throughout diastole. A 9x9 pixel median filter was applied to exclude outliers.

In each temporal frame, peak three-directional flow velocity was determined automatically by the magnitude of the three-dimensional flow vector. The resulting temporal velocity profile was used to determine peak mitral flow and deceleration rate. The individual velocity vectors were then decomposed into the through-plane and in-plane components of flow. By pixel-wise summation of the through-plane velocity components within the masked image at each time point, SV was obtained through temporal integration. The angle of flow was determined from the 50% highest velocities within the mitral valve and calculated as the mean angle between the three-dimensional velocity vector and the through-plane component for these velocities. The principal angle of flow was defined as the angle of flow at time of peak mitral flow. The mean in-plane component of flow within the valve was calculated, and its direction was noted in a standard 17-segment model [17]. The area of mitral flow demarcated a shape similar to an ellipse, and the full width of half maximum (FWHM) of the spatial line profile at the time of peak flow along the long-axis of this shape was measured as a parameter of the flow distribution.

To test the accuracy of the net flow measurements from PC-CMR, flow-derived SV was compared to SV calculated from cine CMR. Our sequence has previously been shown to have excellent accuracy of in-plane velocities, thus, we did not assess further the accuracy of the angular and directional component of flow [14]. Additionally, all CMR post-processing steps and extraction of parameters were performed by a second observer to evaluate interobserver variability.

### *Ex vivo* procedures

Following echocardiography and PC-CMR, the animals were anesthetized with a mixture of 96% O_2_ and 4% O_2_, before the heart was excised in deep surgical anesthesia. Heart weight was obtained before the left ventricle was rapidly dissected and weighted. Finally, lungs were excised and weighted.

### Statistical analysis

Student’s t-tests were performed to compare groups. Comparisons between methods were calculated and presented as Bland-Altman plots, and the mean differences between methods were compared using paired t-tests. Direction of flow was evaluated using contingency tables, with Fisher’s exact post estimation test. Interobserver analysis was performed using the method of Bland and Altman. Statistical analysis was performed using Stata/IC 12.1 (StataCorp, USA). Measurements are shown as mean (standard deviation). Statistical significance was inferred if p<0.05.

## Results

### Pressure overload by AB instigated concentric hypertrophy and heart failure with preserved EF

**Fig 1:**
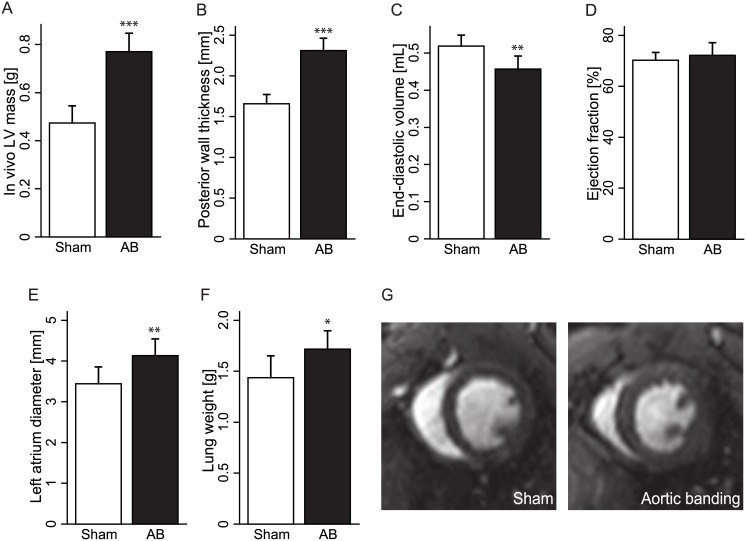
Animal characteristics 6 weeks after aortic banding (AB) or sham procedure. (A) *In vivo* left ventricular (LV) mass. (B) Posterior wall thickness in diastole. (C) End-diastolic volume. (D) Ejection fraction. (E) Left atrium diameter. (F) *Ex vivo* lung weight. (G) Representative cardiovascular magnetic resonance images of concentric hypertrophy in a sham (left) and aortic banding (right) rat. Values are presented as mean ± standard deviation. * indicate p<0.05, ** indicate p<0.01, *** indicate p<0.001 compared to sham.

AB animals presented evidence of concentric hypertrophy, with increased LV mass and LV wall thicknesses, and decreased LV EDV (Fig 1A–1C). *Ex vivo* examinations showed a 58.8% increase in LV weight and a 53.7% increase in heart weight in AB animals compared to sham (Table 1). While maintaining a normal EF (Fig 1D), AB animals showed signs of heart failure, including increased left atrium diameter and lung weight compared to shams (Fig 1E and 1F). A representative mid-ventricular short axis end-diastolic CMR frame is shown for both sham and AB, depicting the concentric hypertrophy seen in AB when compared to sham (Fig 1G).

**Table 1:** Animal characteristics at 6 weeks after aortic banding (AB) or sham procedure.

	Sham	AB
**Count**	7	7
***Cardiovascular magnetic resonance***		
** Heart rate [beats/min]**	399 (20)	397 (29)
** Stroke volume [μL]**	364.1 (26.7)	330.2 (38.7)
** Cardiac output [mL/min]**	145.7 (15.4)	130.4 (12.2)
***Echocardiography***		
** Heart rate [beats/min]**	422 (24)	398 (38)
** Left ventricular dimension, diastole [mm]**	6.7 (0.4)	6.5 (0.7)
** Left ventricular dimension, systole [mm]**	3.2 (0.4)	2.9 (0.4)
** Fractional shortening [%]**	51.6 (4.6)	55.2 (4.4)
** Velocity across stenosis [m/s]**	-	6.9 (1.3)
***At sacrifice***		
** Body weight [g]**	377.9 (21.3)	370.3 (18.4)
** Left ventricular weight [g]**	0.68 (0.10)	1.08 (0.05)***
** Heart weight [g]**	1.08 (0.19)	1.66 (0.17)***

Values are presented as mean (SD).

*** indicate p<0.001 compared to sham

### PC-CMR portrayed temporal and spatial evolution of mitral flow

**Fig 2:**
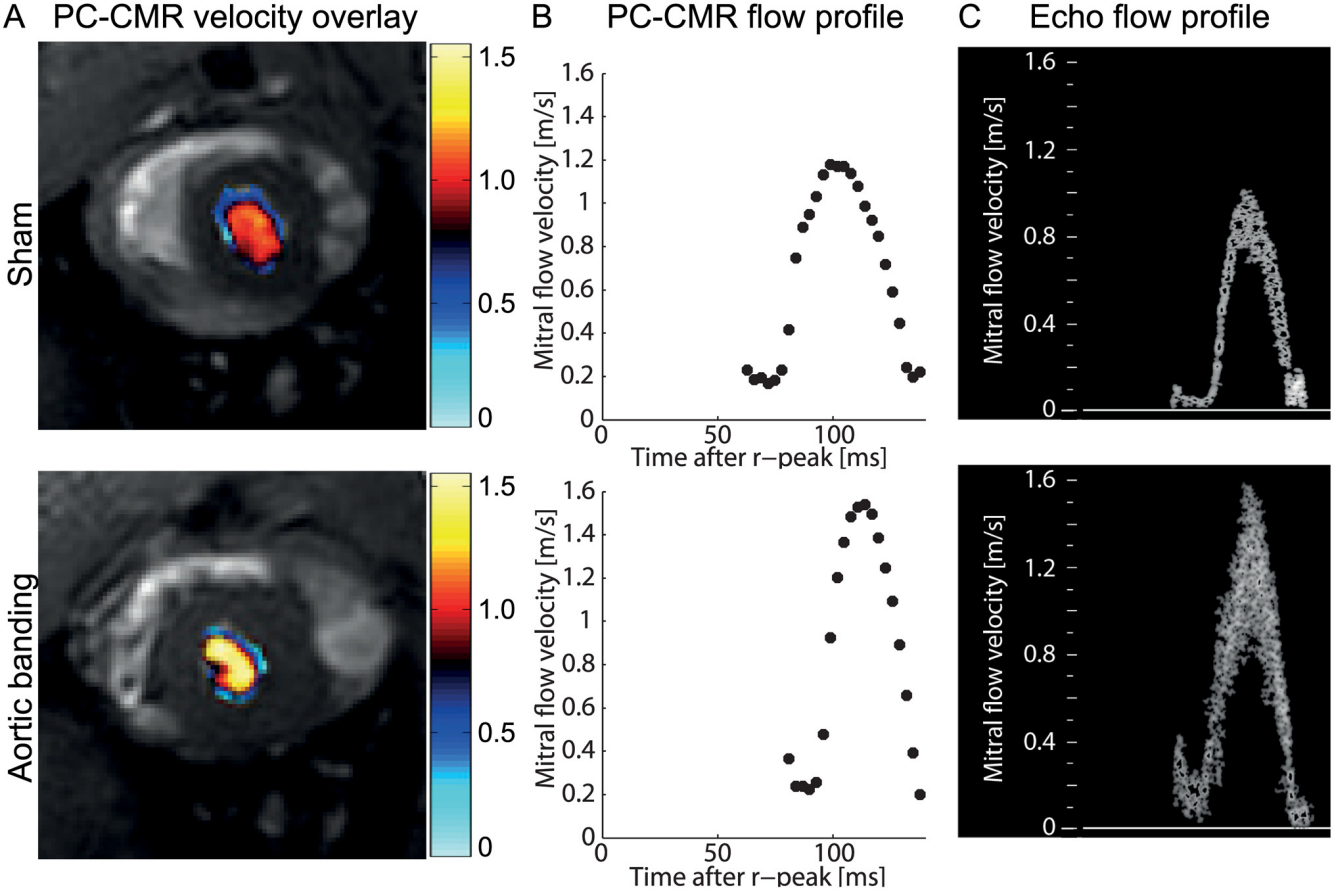
Detection of mitral flow using phase-contrast cardiovascular magnetic resonance (PC-CMR) and echocardiography. (A) PC-CMR mitral flow velocity data overlaid on the magnitude image of the mitral flow in a sham and aortic banding rat. (B) Temporal PC-CMR flow profile during the cardiac cycle in the same animals. (C) Corresponding echocardiographic (echo) Doppler profile of mitral filling.

Spatially resolved mitral flow signals were obtained throughout the diastole by PC-CMR (Fig 2A). The temporal resolution of the PC-CMR was sufficiently high, above 350 Hz, to capture the temporal mitral flow profile (Fig 2B) in a similar fashion as echocardiography (Fig 2C).

### PC-CMR detected AB induced alterations in the mitral flow profile

**Fig 3:**
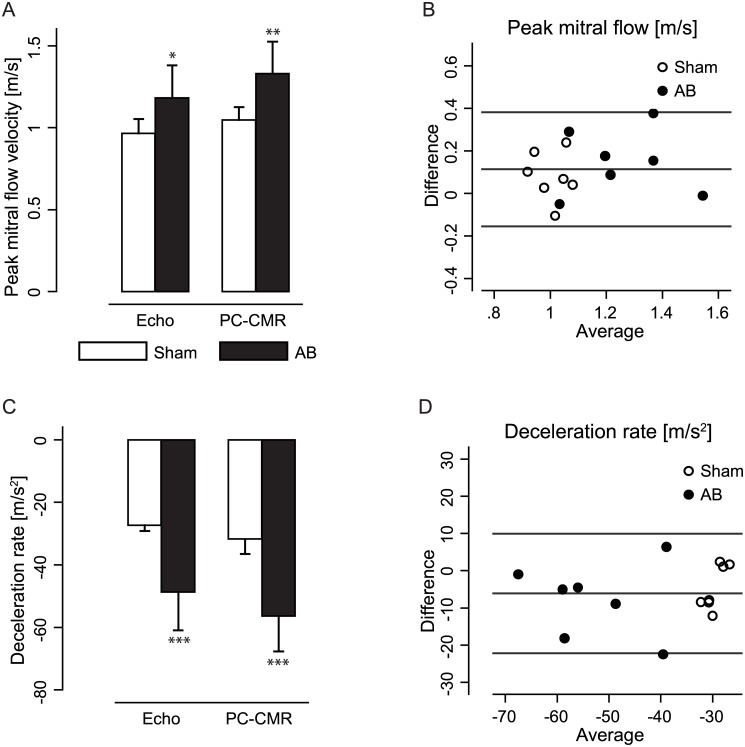
Peak mitral flow and deceleration rate in aortic banding (AB) and sham rats. (A) Peak mitral flow velocity in AB and sham operated rats, using both echocardiography (echo) and phase-contrast cardiovascular magnetic resonance (PC-CMR). (B) Bland-Altman plot describing the difference between echo and PC-CMR in measuring peak mitral flow. (C) Mitral flow deceleration rate in AB and sham rats using echo and PC-CMR. (D) Bland-Altman plot describing the difference between echo and PC-CMR in measuring mitral flow deceleration rate. Values are presented as mean ± standard deviation. * indicate p<0.05, ** indicate p<0.01, *** indicate p<0.001 compared to sham within the corresponding method.

Peak mitral flow was higher in AB compared to sham, as seen with both echocardiography and PC-CMR (Fig 3A), accompanied by an increased deceleration rate of the mitral flow signal in AB compared to sham (Fig 3C). Both peak mitral flow and deceleration rate were measured to be higher with PC-CMR than with pulsed wave Doppler echocardiography when comparing the two methods pairwise in all animals (p = 0.007 and p = 0.014, respectively; Fig 3B and 3D).

### PC-CMR provided new insight into the direction and distribution of mitral flow

**Fig 4:**
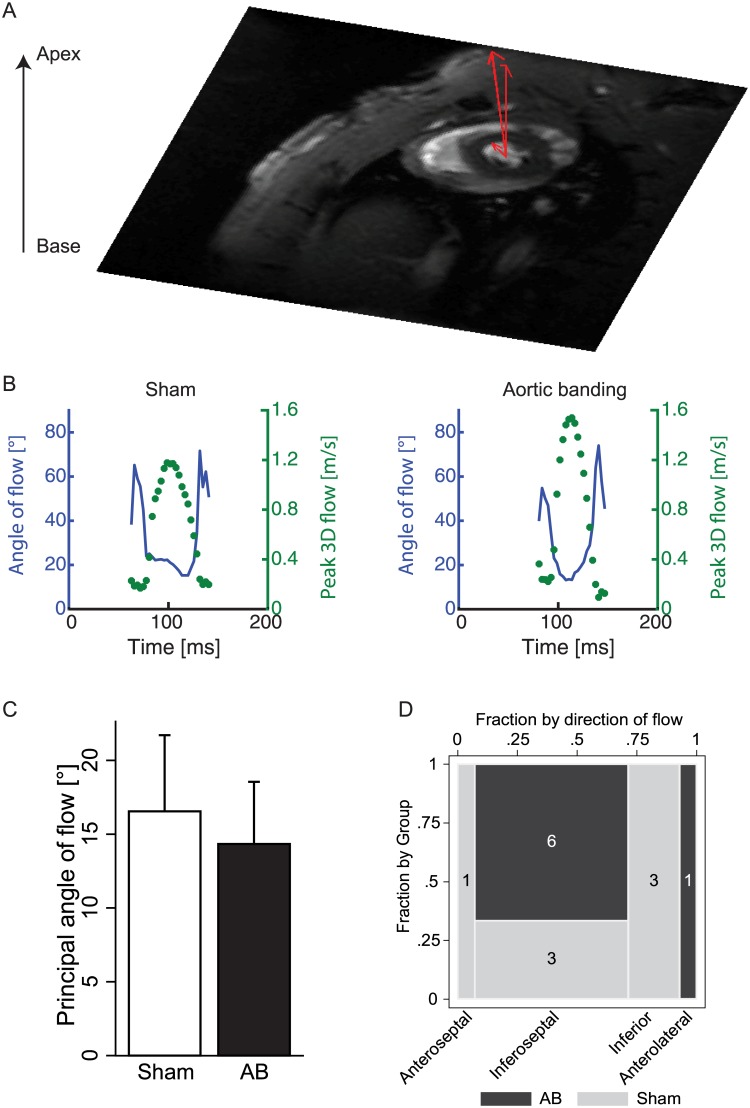
Flow distribution and direction of flow. (A) Three-directional velocity data in the mitral valve, decomposed into the through-plane and in-plane component. (B) Mitral inflow angle (solid, blue line) overlaid with the peak mitral flow profile (dashed, green) during the duration of diastole in a sham and banded animal. (C) The principal angle of flow between the three-directional velocity vector and the through-plane component in sham and the aortic banding (AB) rats. (D) Mosaic plot of the in-plane direction of flow in sham and AB rats describing inferoseptal and/or inferior direction of flow in 12 out of 14 animals. The number in each box indicates the number of animals that display this particular direction of mitral flow. Values are presented as mean ± standard deviation.

Velocity-encoding in all three directions provided information on both through-plane and in-plane directional flow (Fig 4A). Observation of the flow angle throughout the cardiac cycle displayed substantial temporal variation (Fig 4B). While all animals were subject to some degree of temporal variation of flow angle, no pattern was observed within the disease groups. At time of peak mitral flow, the principal angle displayed an average 15.4° shift relative to the plane normal, and was not significantly different between AB and sham animals (Fig 4C). The peak flow was directed inferoseptal or inferior within the plane (12 out of the 14 cases; Fig 4D). The spatial size of the flow distribution was similar in AB and sham (3.98±0.35mm vs. 4.35±0.59mm, p = 0.17).

### PC-CMR provided an accurate evaluation of mitral flow

**Fig 5:**
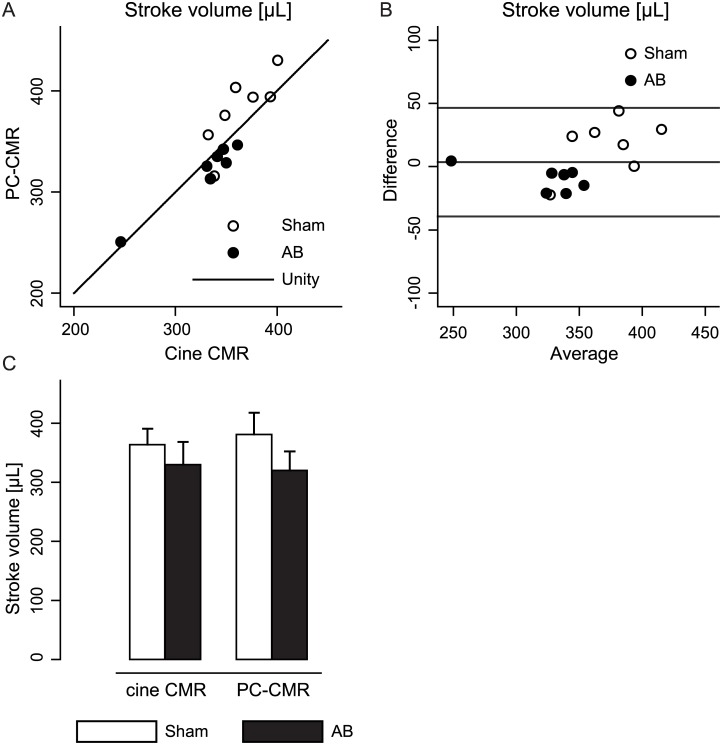
Accuracy of phase-contrast cardiovascular magnetic resonance (PC-CMR) based measurements by evaluation of stroke volume (SV). (A) Scatter plot of stroke volume from PC-CMR and stroke volume evaluated from volumetric (cine) CMR in sham and aortic banding (AB) rats. (B) Bland-Altman plot describing the difference in stroke volume measurements from PC-CMR and cine CMR. (C) Comparison between sham and AB of stroke volume assessed by PC-CMR or cine CMR. Values are presented as mean ± standard deviation.

There was no significant difference in measured SV between PC-CMR and cine CMR (p = 0.54, Fig 5B and 5C). The association between the measurements approached a near unity relationship with mean difference 3.6μL (confidence interval -8.8μL to 16.0μL) and limits of agreement ±42.9μL (an approximate 12.4% discrepancy, Fig 5).

### PC-CMR displayed low interobserver variability

**Fig 6:**
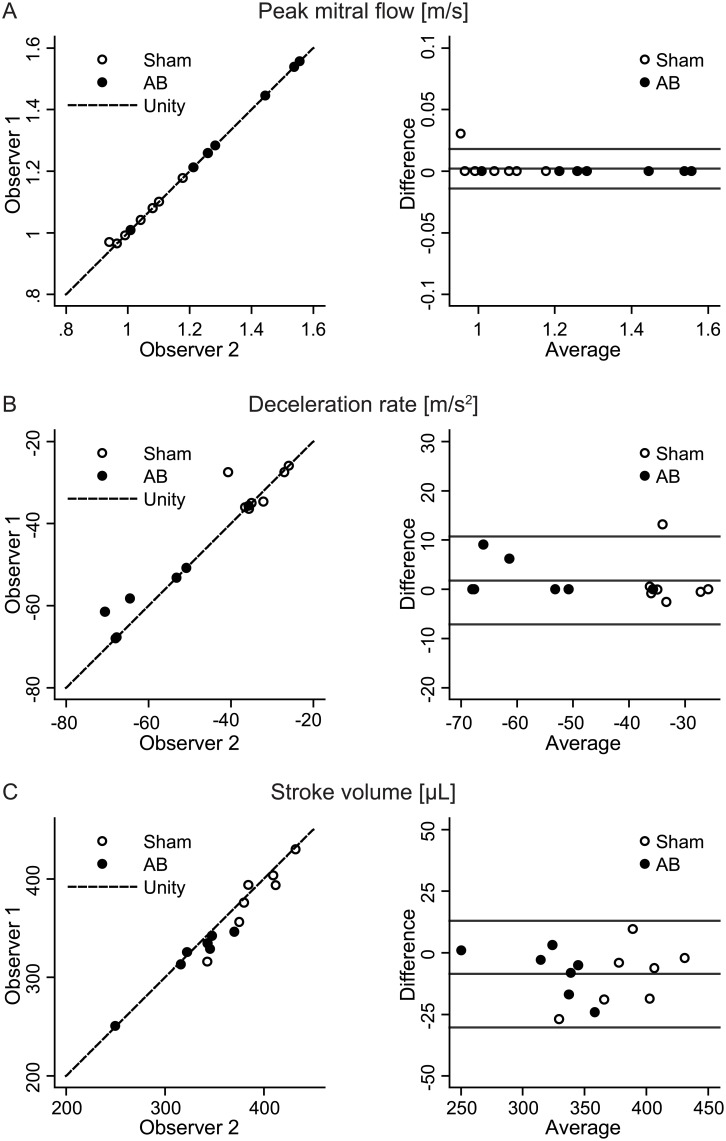
Interobserver variability of PC-CMR mitral flow detection. (A) Scatterplot and Bland-Altman comparison of peak flow velocity measurements from two independent observers in sham and aortic banding (AB) rats. (B) Corresponding interobserver analysis of deceleration rate. (C) Corresponding interobserver analysis of PC-CMR derived stroke volume.

Comparison of PC-CMR parameters between observers showed a close relationship for all variables (slopes not significantly different from 1.0 and R^2^>0.9; Fig 6). The Bland-Altman analysis of peak flow indicated that 95% limits of agreement was 0.002±0.016m/s (Fig 6A). Likewise, limits of agreement for deceleration rate was 1.8±8.9m/s^2^ (Fig 6B), and for stroke volume -8.6±21.7μL (Fig 6C). We concluded that PC-CMR measurements of peak flow, deceleration rate and flow-derived SV consistently provided similar results between observers.

## Discussion

This is the first study to evaluate mitral flow in rodents using PC-CMR. We show that PC-CMR successfully describes the three-directional mitral flow with high spatial and temporal resolution, displaying increased mitral flow and deceleration rate in banded rats compared to sham. PC-CMR offered unique data on flow direction, enhancing the assessment of mitral blood flow in small animals compared to conventional echocardiography. The measurements were validated by comparison with cine CMR, showing that the volume of flow through the mitral valve matched left ventricular stroke volume.

Inspired by the high prevalence of diastolic heart failure, or heart failure with preserved ejection fraction, in the patient population, many basic experimental studies are currently being performed aimed at understanding the mechanisms underlying this disease and allowing for development of novel treatment strategies. More accurate non-invasive methods for evaluation of diastolic function are needed, allowing longitudinal evaluation of cardiac function [18,19]. In this context, the presented PC-CMR based evaluation of mitral blood flow offers new research opportunities.

The three-directional velocity encoding protocol employed in this study allows detection of true velocity independent of any angle between the imaging plane and direction of flow. We present evidence of a septal and/or inferior in-plane direction of mitral flow in most cases, with a 15° tilt compared to the atrioventricular plane normal, and a substantial temporal variation throughout diastole. The occurrence of directional change of flow with progression of disease or in response to pharmaceutical interventions warrants further investigations. In other studies, especially clinical, a unidirectional velocity encoding approach is most common [6,20]. Obvious disadvantages with this unidirectional technique present themselves when the velocity vector is not perpendicular to the imaging plane. Also, three-dimensional imaging with three-directional velocity mapping, commonly referred to as 4D flow, has been performed to assess cardiovascular blood flow in detail [21,22]. Our method is a compromise between these two encoding strategies, employing three-directional velocity encoding in a single imaging slice. Both three-directional methods and unidirectional encoding techniques have previously been compared in the human carotid artery, where unidirectional encoding showed severe underestimation of flow [23]. In the present study, we show that the direction of mitral flow with respect to the atrioventricular plane varies within the animal throughout the cardiac cycle, as well as between animals, emphasizing the need for three-directional flow encoding.

Recently, the 4D flow technique was applied to measure aortic flow in mice [24], potentially allowing quantification of both inflow and outflow in the heart during the complete cardiac cycle. Although our approach is restricted to three-directional measurements within a predefined plane, it offers a 2.5 times better temporal resolution than the current implementation in rodents. Considering the characteristics of the mitral flow profile observed in the rat heart (Fig 2), including fast rate of filling combined with a high heart rate, we believe that the temporal resolution demonstrated in the present study is a minimum requirement to accurately detect true peak flow.

Although we believe the temporal resolution of our sequence is sufficient, there will always be a potential for undersampling the data temporally, compared to for instance echocardiography. However, this low pass effect appears to be of minimal importance in our study, supported by the volume validation with cine CMR. Another limitation with our technique is the use of a static imaging slice. Although we aim to position the slice such that mitral excursion does not contribute significantly to the velocities detected in the slice, a higher accuracy could be achieved by correcting for the mitral plane motion during diastole, for instance using 4D flow measurements or a moving imaging slice trailing the mitral valve. Finally, a limitation with our approach is how the interobserver analysis was only performed post acquisition of the data. However, we believe that our method should be robust to slight differences in slice positioning by evaluating the three-directional velocity and not the through-plane component.

The present study showed significantly higher peak mitral flow velocity and flow deceleration rate with PC-CMR compared to echocardiography. Still, echocardiography and PC-CMR gave the same qualitative relationship between sham and AB flow parameters. Since conventional echocardiography relies on proper angle correction and precise positioning of the finite sampling volume, we believe that PC-CMR better describes the true characteristics of mitral flow. Our view is supported by the close correspondence between the volume measurements from PC-CMR and the stroke volume from cine CMR, approaching a near unity relationship (Fig 5A).

## Conclusions

For the first time, we show that PC-CMR offers accurate evaluation of three-directional mitral blood flow. The method successfully detects alterations in the mitral flow pattern, in response to cardiac disease and provides novel insight into the characteristics of mitral flow.

## Supporting Information

S1 Data Supporting data file. Data file presenting all *in vivo* and *ex vivo* measures underlying the findings presented in this paper.
(XLS)
